# Hydrostatic pressure mediates epithelial-mesenchymal transition of cholangiocytes through RhoA/ROCK and TGF-β/smad pathways

**DOI:** 10.1371/journal.pone.0300548

**Published:** 2024-04-05

**Authors:** Mahmoud Osman Khalifa, Chen Yan, Yong Chai, Kosei Ito, Shou-Hua Zhang, Tao-Sheng Li

**Affiliations:** 1 Department of Stem Cell Biology, Atomic Bomb Disease Institute, Nagasaki University, Nagasaki, Japan; 2 Department of Molecular Bone Biology, Graduate School of Biomedical Sciences, Nagasaki University, Nagasaki, Japan; 3 Department of Anatomy and Embryology, Veterinary Medicine, Aswan University, Aswan, Egypt; 4 Department of General Surgery, Jiangxi Provincial Children’s Hospital, Nanchang, Jiangxi, China; Macau University of Science and Technology, HONG KONG

## Abstract

Biomechanical cue within the tissue microenvironment is known to play a critical role in regulating cell behaviors and maintaining tissue homeostasis. As hydrostatic pressure often increases in biliary system under pathological states, we investigated the effect of the moderate elevation of the hydrostatic pressure on biliary epithelial cells, especially on the epithelial-mesenchymal transition (EMT). Human intrahepatic biliary epithelial cells were loaded to hydrostatic pressure using a commercial device. We found that loading the cells to 50 mmHg hydrostatic pressure induced obvious morphological changes and significantly upregulated vimentin, ZEB1, and pSmad2/3, fibronectin, and collagen 1α. All changes induced by hydrostatic pressure loading were effectively mitigated by either ROCK inhibitor (Y-27632) or ALK5 inhibitor (SB-431542). Our *in vitro* experimental data suggests that hydrostatic pressure loading induces EMT of cholangiocytes through RhoA/ROCK and TGF-β/Smad pathways. Elevated hydrostatic pressure in biliary duct system under pathological states may promote the biliary epithelial cells shifting to profibrotic and mesenchymal characteristics.

## Introduction

The cholangiocytes are a heterogeneous population of epithelial cells that line the biliary tree forming a canicular duct. It has been reported about the phenotypic plasticity of cholangiocytes in response to the alternations of surrounding tissue microenvironment [1–3]. Obstructive cholestasis and other inflammatory conditions, such as biliary cirrhosis and sclerotic cholangitis occur in the interstitial space altering tissue homeostasis [4]. Recent studies have demonstrated that cholangiocytes can contribute to fibrogenesis and the evolution of an inflammatory signaling cascade, especially during the tracing of acute liver injury resulting from cholestasis [4, 5]. Most of mechanical stress affects cell plasticity can enhance direct ion channels in addition to receptor-legend complex formation for cell signaling pathway in response to the surrounding microenvironment [6, 7]. It has also been reported that intrahepatic biliary epithelial cells contribute in a part to the epithelial-mesenchymal transition (EMT) process during liver fibrosis [8, 9]. Therefore, targeting the key molecules of mechanosensing and mechanotransduction to blockade the biological alternations of cholangiocytes may provide a novel therapeutic approach on hepatobiliary diseases.

EMT is well known as the phenotypic change of epithelial cells to cells with mesenchymal properties, which is characterized by the upregulation of mesenchymal markers (e.g., vimentin, N-cadherin, fibronectin) and the downregulation of epithelial markers (e.g., cytokeratin, E-cadherin, β-catenin) [10, 11]. Many growth factors and inflammatory cytokines (e.g., TGF-β, PDGF, IL-1β) have been demonstrated to promote EMT by activating cell signaling pathways to initiate the transcription of EMT-associated factors (TWIST1, ZEB1/2, SNAIL1/2) [12].

Biomechanical stresses that arise from the extracellular matrix (ECM) stiffness and mechanical forces such as interstitial fluid pressure in the tissue microenvironment play critical roles in regulating cell behaviors and tissue homeostasis [13]. It is known about the dynamic changes of biomechanical cues within biliary duct system and the surrounding tissues under pathological states such as the retrograde biliary duct obstruction. As the alterations of biomechanical cues under pathological states will be very complicated, it is difficult to evaluate the precise role and relevant molecular mechanism of biomechanical forces in mediating the development and progression of hepatobiliary diseases.

In this study, we proposed to investigate whether biomechanical stresses promote EMT of cholangiocytes by *ex vivo* approach. To mimic the elevated hydrostatic pressure under the retrograde biliary duct obstruction, one of the most common pathogenic states, we simply loaded primary human biliary epithelial cells to 50 mmHg pressure using a commercial device, and then evaluated the biological properties of the cells.

## Materials and methods

### Cell culture

Human intrahepatic biliary epithelial cells were purchased from the Sciencell (Catalog #5100). The cells were suspended in the recommended epithelial medium (EpiCM, Catalog #4101) that consists of 500 ml of basal medium, 10 ml of fetal bovine serum (FBS, Cat. No. 0010), 5 ml of epithelial cell growth supplement (EpiCGS, Cat. No. 4152), and 5 ml of penicillin-streptomycin antibiotic solution (Cat. No. 0503) with a seeding density of 20,000 cells/ml and cultured under 37°C in a 5% CO_2_ incubator.

### Hydrostatic pressure stimulation

When the cells were grown to 50–70% confluence, we randomly kept the cells at following conditions: 1) under normal atmospheric condition in ordinary incubator (AP group); 2) under 50 mmHg hydrostatic pressure induced by a commercial device as described previously [14] (PP group); 3) under 50 mmHg hydrostatic pressure with the addition of Rho-associated protein kinase (ROCK) inhibitor Y-27632 (10 μM; ATCC, ACS-3030^™^, USA) in medium (PP+Y27 group); and 4) under 50 mmHg hydrostatic pressure with the addition of SB-431542 (10 μM; MCE, HY-10431, Japan), an inhibitor to activin receptor-like kinase 5 (ALK5, also known as TGF-β type I receptor) (PP+SB43 group).

### Cell viability and cell morphological analyses

The number and viability of cells were evaluated at 6 and 48 hr after treatments. Briefly, the cells were harvested, stained with 0.4% trypan blue (BIO-RAD, Cat.1450013), and counted by TC20 automated cells counter (BIO-RAD, USA). Cell morphology was detected under phase contrast microscopy, and the ratio of longitudinal to the transverse axis of cells was also measured using Image J software.

### Immunofluorescence staining

The cells were seeded on clean glass coverslips and cultured at different conditions as described above. Cells were fixed with 4% paraformaldehyde in PBS and permeabilized with 0.3% Triton X-100 (Sigma-Aldrich) in PBS solution for 15 min. After blocking with 5% bovine serum albumin in PBS for 1 hr, the cells were incubated with the primary antibodies against vimentin (Catalog #5741), ZEB1 (Catalog #70512), pSmad2/3 (Catalog #8828), fibronectin (Catalog #26836), and COL1A1 (Catalog #66948) overnight at 4°C, and then followed by incubating with the corresponding secondary antibodies in dark for 1 hr at room temperature. Finally, the cells were mounted with VECTASHIELD Hardset Antifade mounting medium with DAPI (H-1500, Funakoshi). Images were taken with a confocal fluorescent microscope (Olympus FV10i). The fluorescence intensity of staining was evaluated by Image J software.

### Statistical analysis

All quantitative data are presented as the mean ± standard deviation from three independent experiments. Statistical analyses were done either with one- or two-way analysis of variance and followed by Tukey’s multiple comparisons using GraphPad Prism software (Prism version 8, San Diego, California USA). The significant differences between groups were considered at *p* < 0.05.

## Results

**Fig 1:**
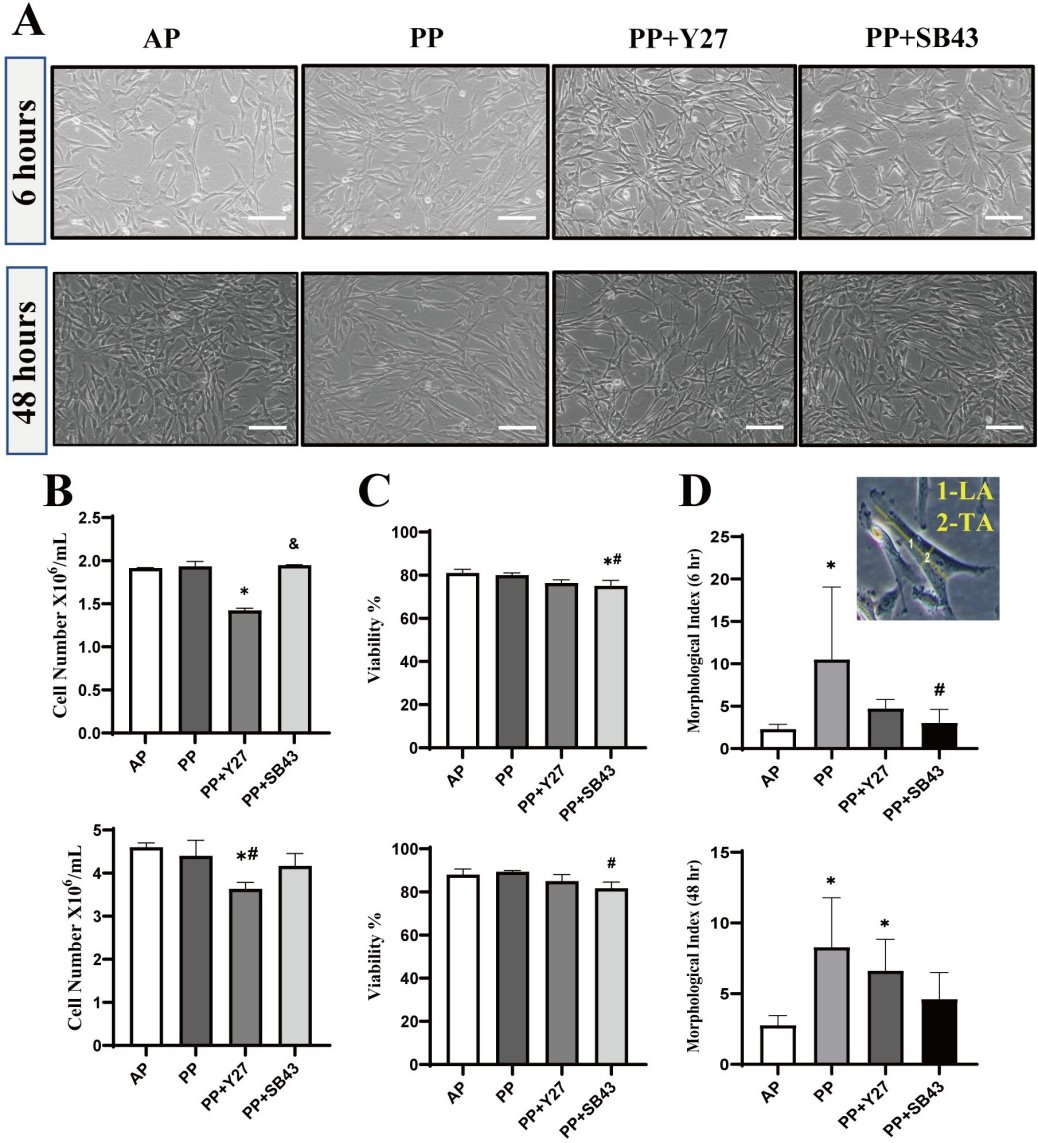
Cell viability and morphological changes under 50 mmHg hydrostatic pressure loading and ROCK and ALK5 inhibitor treatment. (**A**) Representative phase-contrast images show the morphology of cells (Scale bars: 200 μm). Quantitative data on cell number (**B**) and cell viability (**C**) are shown. (**D**) Morphological index, the ratio of longitudinal to the transverse axis. LA: Longitudinal axis; TA: Transverse axis. All data are presented as mean ± SD from three independent experiments. AP: atmospheric pressure; PP: positive pressure; PP+Y27: positive pressure plus ROCK inhibitor Y-27632. PP+SB43: positive pressure plus ALK5 inhibitor SB-431542. *p < 0.0001 *vs*. AP group; ^#^*p* < 0.0001 *vs*. PP group; ^&^*p* < 0.0001 *vs*. PP+SB43 group in all time points, 6 and 48 hours.

EMT of cells is a biological mechanism usually accompanied by morphological changes [15]. Under phase contrast microscopy, we noticed that many cells acquired fibroblast-like shape under the loading to 50 mmHg hydrostatic pressure (**Fig 1A**). The cell number was counted a little less in the PP+Y27 group than other groups (**Fig 1B**). However, cell viability was lower in PP+SB43 group than other groups (**Fig 1C**). By measuring the ratio of the longitudinal to transverse axis, cells in the PP group showed significantly higher value than the control group at 6 and 48 hr (**Fig 1D**), and some cells in PP group showed a clear duct-like structures (**Fig 1D**). These morphological changes of cells induced by hydrostatic pressure loading were effectively attenuated by either ROCK inhibitor or ALK5 inhibitor.

**Fig 2:**
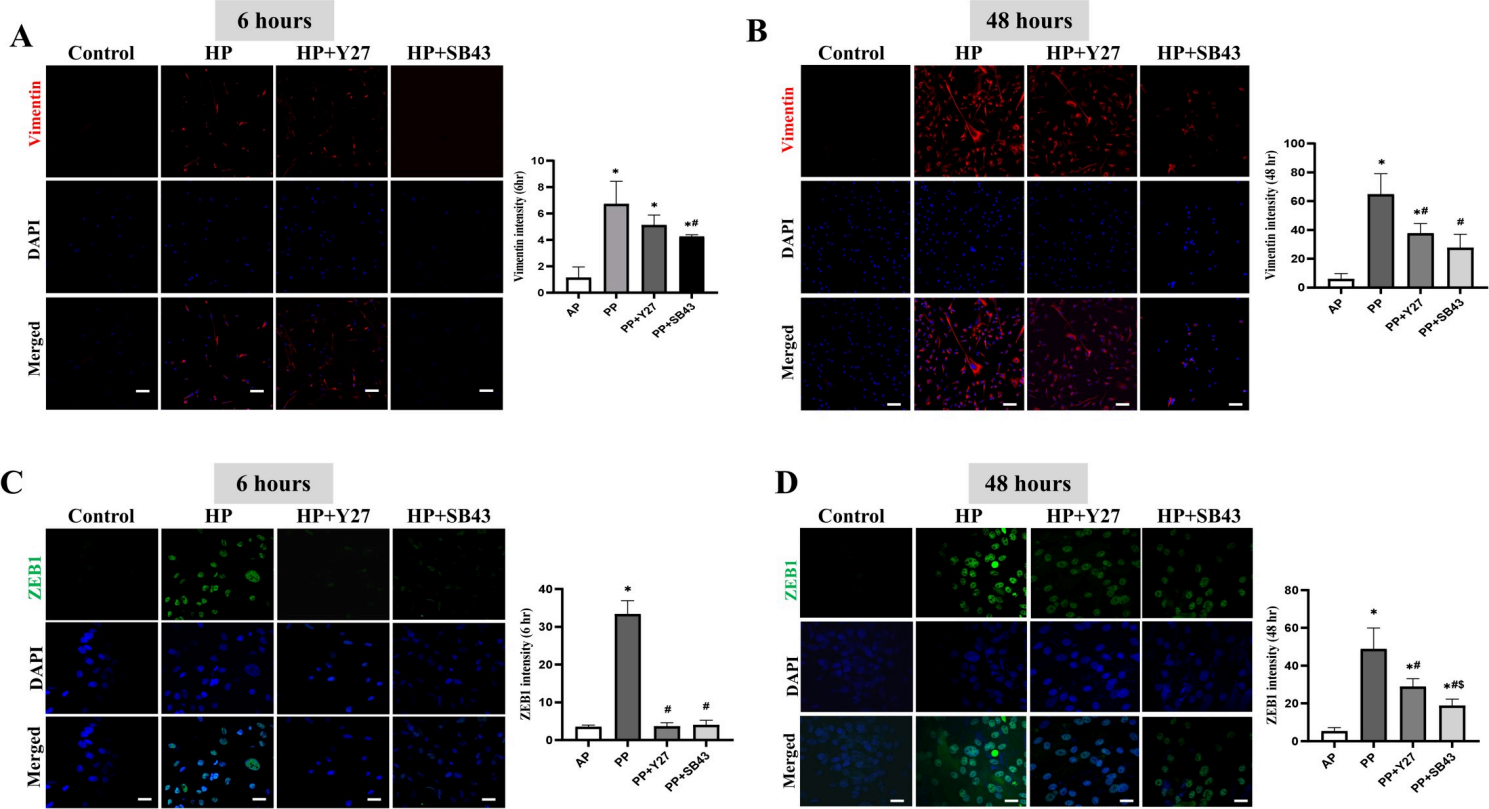
The expression of vimentin and ZEB1 in cholangiocytes. Immunofluorescent images of vimentin (left panel) and quantitative data (right side) at 6 hours (**A**) and 48 hours (**B**) after treatments (Scale bars in images: 20 μm). Immunofluorescent images of ZEB1 expression (left panel) and quantitative data (right side) at 6 hr (**C**) and 48 hr (**D**) after treatment (Scale bars in images: 50 μm). All data are presented as mean ± SD from three independent experiments. AP: atmospheric pressure; PP: positive pressure; PP+Y27: positive pressure plus ROCK inhibitor Y-27632. PP+SB43: positive pressure plus ALK5 inhibitor SB-431542. *p < 0.0001 *vs*. AP group; ^#^*p* < 0.0001 *vs*. PP group; ^$^*p* < 0.0001 *vs*. PP+SB43 group.

We next investigated the common hallmarks of epithelial and mesenchymal characterization undergo EMT. Compared to the control, cells subjected to 50 mmHg hydrostatic pressure resulted in a robust enhancement on the expression of vimentin at 6 and 48 hr (**Fig 2A & 2B**) but was partially cancelled by either ROCK inhibitor or ALK5 inhibitor. Consistently, ZEB1, one of core EMT-associated transcription factors was drastically upregulated in cells with 50 mmHg hydrostatic pressure loading for 6 and 48 hr (**Fig 2C & 2D**) but was effectively attenuated by either ROCK inhibitor or ALK5 inhibitor.

**Fig 3:**
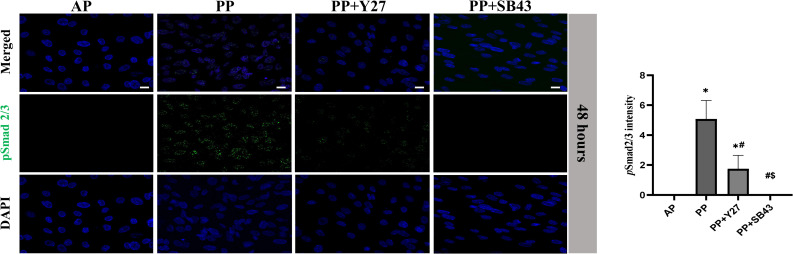
The expression of pSmad2/3 in cholangiocytes. Representative images (left) and quantitative data (right bar graph) show the immunostaining of the pSmad2/3 at 48 hr after treatments (Scale bars: 50 μm). All data are presented as mean ± SD from three independent experiments. AP: atmospheric pressure; PP: positive pressure; PP+Y27: positive pressure plus ROCK inhibitor Y-27632. PP+SB43: positive pressure plus ALK5 inhibitor SB-431542. *p < 0.0001 *vs*. AP group; ^#^*p* < 0.0001 *vs*. PP group; ^$^*p* < 0.0001 *vs*. PP+SB43 group.

TGF-β/Smad signaling pathway is strongly involved in the process of EMT induction [10, 16]. Immunostaining showed that the phosphorylated Smad2/3 (pSmad2/3) were robustly upregulated in cells at 48 hr after 50 mmHg hydrostatic pressure loading (**Fig 3**). The hydrostatic pressure-induced enhancement of pSmad2/3 was partially canceled by ROCK inhibition and almost completely canceled by ALK5 inhibition (**Fig 3**).

**Fig 4:**
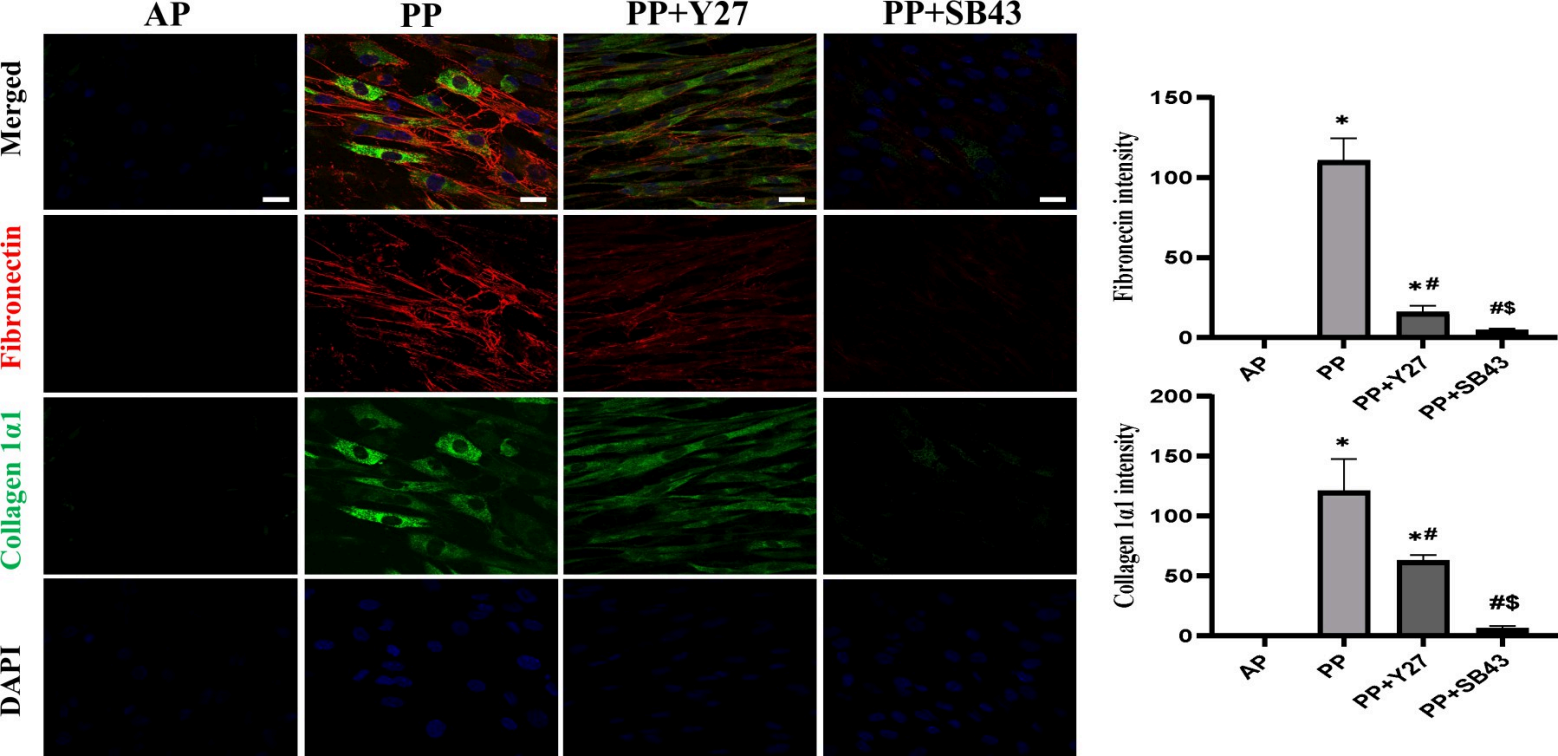
The expression of fibronectin and collagen 1α in cholangiocytes. Double immunostaining analysis on the expression of collagen 1α (Green) and fibronectin (Red) in cholangiocytes at 48 hr after treatments (Scale bars: 20 μm). All data are presented as mean ± SD from four independent experiments. AP: atmospheric pressure; PP: positive pressure; PP+Y27: positive pressure plus ROCK inhibitor Y-27632. PP+SB43: positive pressure plus ALK5 inhibitor SB-431542. *p < 0.0001 *vs*. AP group; ^#^*p* < 0.0001 *vs*. PP group; ^$^*p* < 0.0001 *vs*. PP+SB43 group.

As EMT is accompanied by ECM deposition, we compared the expression of collagen 1α and fibronectin in the cells among groups (**Fig 4**). The loading of cells to 50 mmHg hydrostatic pressure for 48 hr dramatically induced the expression of collagen 1α and fibronectin, although there was almost negatively detected in control (**Fig 4**). Either ROCK inhibitor or ALK5 inhibitor effectively mitigated the enhancement of collagen 1α and fibronectin in cells induced by 50 mmHg hydrostatic pressure loading (**Fig 4**).

## Discussion

The cellular plasticity is commonly observed in cancer cells, but also recognized in normal tissue cells. EMT is a biological process can be seen in embryonic gastrulation, wound healing, and cancer progression to metastasis [17, 18]. Many studies have demonstrated that the EMT process is closely associated with fibrogenesis in the liver and the kidneys [19, 20]. EMT of renal epithelium can be involved in interstitial fibrosis [21]. Obstructive cholestasis is known to induce liver fibrosis. We have also recently reported that, in response to ureteral obstruction, the resident mesenchymal stem-like cells change into profibrotic phenotype to contribute renal fibrogenesis [22]. Moreover, targeting mechanotransduction using very low dose losartan has been demonstrated to effectively mitigate liver fibrosis in mice receiving partial ligation of inferior vena cava [23]. Therefore, biomechanical stresses, organ fibrogenesis, and EMT of tissue cells are likely links to each other.

The interstitial fluid hydrostatic pressure is generally increased in almost all tissues/organs under pathological conditions. In obstructive cholestasis and other inflammatory conditions, elevated hydrostatic pressure in the interstitial space and biliary tract occurs in the live. The biomechanical cues within tissue microenvironment are well known to regulate the biological behaviors of tissue cells [24–26]. However, it has been poorly understood about the precise role of altered mechanical forces on the progression of diseases, because it is difficult to distinct the direct effect of altered mechanical forces from other factors such as the changes of inflammatory cytokines and growth factors in the tissue microenvironment under pathological states.

Using *ex vivo* approach to simplify the very complicated factors of biomechanical cues in tissue microenvironment, we have recently demonstrated that elevated hydrostatic pressure promotes cancer cell metastasis by stabilizing HIF-1α expression [27], induces profibrotic properties in hepatic stellate cells *via* the RhoA/ROCK signaling pathway [14], activates profibrotic transcription of atrial stromal cells *via* TGF-β signal pathway [28], and affects lymphocyte activation *via* complex mechanisms [29]. Similarly, we herein investigated whether elevated hydrostatic pressure promotes EMT of cholangiocytes *in vitro*. In a healthy liver, the hydrostatic pressure keeps at very low level in the interstitial space (near to zero) and biliary tract (<10 mmHg), but the maximum pressure in common bile duct has been measured as 21–68 mmHg in patients with gallstones [30]. Combined with our preliminary investigation, we used 50 mmHg for experiment in this study. We found that the loading of primary human biliary epithelial cells to 50 mmHg significantly upregulated vimentin, fibronectin, and collagen, suggesting the shifting into mesenchymal and profibrotic phenotypes.

It has been well documented that ZEB1, TWIST1, SNAIL, and Slug are the core transcription factors for EMT process [12]. ZEB1 is a key factor for cell fate determination, including cell plasticity and cancer cell metastasis [31]. The loading of biliary epithelial cells to 50 mmHg also enhanced the expression of ZEB1, but the crucial role of ZEB1 in EMT process is asked to further confirm by interfering experiments.

As the pioneer regulatory pathway of mechanotransduction, RhoA/ROCK signaling initiates various downstream cascades to mediate the biological behaviors of cells in response to the dynamic changes of biomechanical forces [32]. Miyano et al., [33] has recently demonstrated that the induction of hyperosmotic stress to tubular epithelial cells can lead to upregulation of EMT biomarkers and related biomarkers through the activation of mechanotransduction. TGF-β/Smad signaling is known as the most important pathway in EMT process [34]. Therefore, we used ROCK inhibitor Y-27632 and ALK5 inhibitor SB-431542 to understand the mechanism involving in the mechanical stress-induced EMT. As expected, either ROCK inhibitor or ALK5 inhibitor effectively mitigated but not completely blocked the EMT of biliary epithelial cells induced by 50 mmHg hydrostatic pressure. Although other signal pathways may also be involved in, it seems that elevated hydrostatic pressure promotes cholangiocytes to acquire mesenchymal and profibrotic properties mainly through RhoA/ROCK and TGF-β/Smad pathways.

This study has several limitations. The first, we only used 50 mmHg for experiment, but the response of cells can be largely varied in response to different mechanical pressure. The second, all experiments were performed under 2D cell culture condition, that is completely differed from the biomechanical cues within the tissue microenvironment of hepatobiliary diseases in which may accompanying the change of ECM stiffness. Otherwise, due to the problem of enhanced expression of mesenchymal markers in immortalized cells, we used primary human intrahepatic biliary epithelial cells for experiment. As these primary cells have very limited proliferative property, it is difficult to expand enough cells to isolate proteins for Western blot to further confirm our findings from immunostaining analysis.

In conclusion, our *in vitro* investigations provided clear evidence on the mechanical stress-induced EMT of biliary epithelial cells. As the elevated hydrostatic pressure or altered ECM stiffness, may change the biological behaviors of cholangiocytes, such as the EMT via mechanotransduction pathways. Therefore, targeting key molecules on mechanosensing and mechanotransduction may provide be a novel therapeutic approach on hepatobiliary diseases.
